# Reciprocal rescue of sensory cell cilia defects by *Cep290* and *Mkks* alleles

**DOI:** 10.1186/2046-2530-1-S1-P94

**Published:** 2012-11-16

**Authors:** H May-Simera, R Rachel, B Choi, T Li, T Friedman, A Swaroop, M Kelley

**Affiliations:** 1 NEI, National Institute of Health, USA; 2 NIDCD, National Institute of Health, USA

## 

Ciliopathies are developmental disorders that arise due to defects in cilia biogenesis and function, and affect various sensory systems including the auditory system. Involvement of multiple syndromic ciliopathy genes, whose protein products are thought to function as macromolecular complexes in both cilia and basal bodies, implicate dynamic regulation of ciliary protein interactions. Mutations in *CEP290* (NPHP6/BBS14), cause several ciliary disorders [Leber congenital amaurosis (LCA), Senior-Loken syndrome, Joubert syndrome, nephronophthisis (NPHP), Meckel-Gruber syndrome (MKS) and Bardet-Biedl syndrome (BBS)]. Little is known about the function of CEP290, or how this protein interacts with other cilia-related proteins complexes. An initial finding of variants of MKKS (also known as BBS6) in LCA patients led to an exploration of epistatic interactions between CEP290 and MKKS. We found that the DSD domain of CEP290, which is deleted in a mouse model (*Cep290rd16*) of LCA, directly interacts with MKKS, and that pathogenic variants of MKKS disrupt this interaction. Mice with either *Cep290rd16/rd16* or *Mkksko/ko* genotypes exhibit structural and functional auditory, photoreceptor, and olfactory deficits. Unexpectedly, *Cep290rd16/rd16;Mkksko/ko* double mutants actually show a degree of functional and/or morphological rescue in all three sensory systems by comparison with either single mutant. Moreover, mice with triple allelic combinations of *Cep290rd16* and/or Mkksko appear more fully rescued than *Cep290rd16/rd16; Mkksko/ko* double mutants. Morphological analysis suggests that improved ciliogenesis forms the mechanistic basis for this functional rescue. Our data demonstrate reciprocal modifier effects between the CEP290 DSD domain and MKKS that provides insight into the regulation of cilia formation and function.

